# Antibody responses after intravaginal immunisation with trimeric HIV-1_CN54_ clade C gp140 in Carbopol gel are augmented by systemic priming or boosting with an adjuvanted formulation

**DOI:** 10.1016/j.vaccine.2010.12.034

**Published:** 2011-02-04

**Authors:** Martin P. Cranage, Carol A. Fraser, Alethea Cope, Paul F. McKay, Michael S. Seaman, Tom Cole, A. Nasir Mahmoud, Joanna Hall, Elaine Giles, Gerald Voss, Mark Page, Neil Almond, Robin J. Shattock

**Affiliations:** a Centre for Infection and Immunity, Division of Clinical Sciences, St George's, University of London, Cranmer Terrace, London SW17 0RE, UK; b CAVD Neutralizing Antibody Laboratory, Beth Israel Deaconess Medical Center, Harvard Medical School, Boston, MA, USA; c Division of Retrovirology, National Institute for Biological Standards and Control, South Mimms, Potters Bar, UK; d GlaxoSmithKline Biologicals, Rixensart, Belgium

**Keywords:** Immunisation, Vagina, HIV-gp140

## Abstract

Optimum strategies to elicit and maintain antibodies at mucosal portals of virus entry are critical for the development of vaccines against human immunodeficiency virus (HIV). Here we show in non-human primates that a novel regimen of repeated intravaginal delivery of a non-adjuvanted, soluble recombinant trimeric HIV-1_CN54_ clade C envelope glycoprotein (gp140) administered in Carbopol gel can prime for B-cell responses even in the absence of seroconversion. Following 3 cycles of repeated intravaginal administration, throughout each intermenses interval, 3 of 4 macaques produced or boosted systemic and mucosally-detected antibodies upon intramuscular immunisation with gp140 formulated in AS01 adjuvant. Reciprocally, a single intramuscular immunisation primed 3 of 4 macaques for antibody boosting after a single cycle of intravaginal immunisation. Virus neutralising activity was detected against clade C and clade B HIV-1 envelopes but was restricted to highly neutralisation sensitive pseudoviruses.

## 1 Introduction

The induction of responses that protect at mucosal portals of virus entry poses a particular problem for vaccine design and development. Nowhere is this more critically highlighted than in the search for an HIV vaccine where prevention of infection at, and/or rapid clearance from, the mucosal surface may be essential for vaccine efficacy. Ideally an effective vaccine would induce virus neutralising activity in the fluids present at susceptible mucosal surfaces such as the lower female genital tract. Despite over 20 years of intensive research, this is proving to be a complex problem with many roadblocks to progress. In part this is due to the particular biology of HIV including (1) the structure of the virus glycoprotein spikes that are largely resistant to the induction and action of neutralising antibodies through conformational masking [1,2], glycan shielding [3,4] and sequence hypervariability [5]; (2) the rapid dissemination of virus from mucosal sites of infection [6–8] and (3) the potential for HIV to evade antibody through intimate cell-to-cell spread (reviewed in Martin and Sattentau [9]). However, significant progress is being made. Examples of broadly reactive virus-neutralising antibodies and their cognate epitopes are increasingly being described [10–12] and significantly, protective efficacy has been reported in macaques against vaginal, oral and rectal challenge with HIV-simian immunodeficiency (SIV) Env-chimeric viruses (SHIVs) following intravenous infusion of neutralising monoclonal antibodies [13–17].

A further significant roadblock to progress, addressed in the study reported here, is how to induce and maintain anti-HIV antibody responses at mucosal surfaces. Not only is there a lack of licensed mucosal adjuvants but there is also the danger of creating additional targets for HIV-infection through the activation and/or recruitment of local T cells, a potential problem highlighted in the STEP IIb clinical trial using recombinant adenovirus 5 (Ad5) vectors [18]. Furthermore, mucosal effector B-cell responses are relatively short-lived. Thus, for pathogens such as HIV, that gain direct access to the immune system, it may be necessary to provide repeated or sustained stimulation of local specific immunity in the absence of generalised inflammation to maintain a protective antibody response. We are addressing this issue in animal models and in women using vaginal immunisation with stable recombinant HIV-1_CN54_ clade C trimeric gp140 produced in CHO cells. A clade C product was chosen based upon the high prevalence of this HIV-1 subtype globally. To minimise the chance of causing local inflammation, the antigen is formulated in a poly-acrylic acid (Carbopol) gel, an excipient licensed for vaginal use in women. Because, in women, the efficiency of vaginal immunisation is influenced by the menstrual cycle [19,20], formulated antigen is administered repeatedly throughout the intermenses interval to ensure exposure at the optimal time. Thus, a single cycle of immunisation consists of 9 exposures intravaginally. We have reported previously that a single cycle of repeated intravaginal administration of this formulation was sufficient to reproducibly induce antibody responses in rabbits [21]. The data, from this pre-clinical vaginal irritancy study, proved the concept that exposure of the female genital tract to non-adjuvanted recombinant HIV gp140 can induce systemic and mucosally-detectable antibodies and showed that the formulation was well tolerated. However, ovulation is coitally-induced in rabbits and the anatomy of the rabbit female genital tract may favour antigen uptake, being markedly different to that of women [22].

Here we have immunised cynomolgus macaques intravaginally with trimeric HIV-1_CN54_ gp140 mixed with Carbopol gel using a protocol identical to that used in a clinical trial run in parallel. Although the present study was not designed for virus challenge, it is important to compare immunogenicity in macaques and humans so that subsequent vaccine efficacy studies with SIV or SHIVs [23] can be fully interpreted. Moreover, this strategy affords the opportunity to iteratively evaluate variations of the vaccine protocol before moving the most promising options to human phase 1 studies and to macaque virus challenge studies. We have used the macaque model to determine the effects of multiple cycles of intravaginal immunisation and the effects of subsequent and prior intramuscular immunisation with trimeric gp140 formulated in the GSK Biologicals AS01 Adjuvant System containing liposomes, monophosphoryl lipid A (MPL) and Quillaja saponaria fraction 21 (QS21) [24,25]. We show that systemic and mucosally-detected IgG and IgA responses are induced in a proportion of animals after repeated vaginal exposure to HIV-1 clade C envelope formulated in a Carbopol gel and were efficiently boosted by subsequent intramuscular immunisation with adjuvanted gp140. Furthermore, intravaginal immunisation could prime, without prior seroconversion, for a memory response revealed by intramuscular immunisation. Reciprocally, a single intramuscular immunisation primed for intravaginal boosting.

## 2 Materials and methods

### 2.1 HIV gp140

A clade C envelope clone p97CN54 was obtained originally from a Chinese patient [26,27] and was made available by H. Wolf and R. Wagner, University of Regensburg, Germany. Trimeric gp140 (gp120 plus ED of gp41), designated as CN54 gp140, was produced in CHO cells as a recombinant product and manufactured to good manufacturing practice specification by Polymun Scientific, Vienna Austria. The fidelity of the product was confirmed by mass spectrometric analysis of tryptic fragments, by the Medical Biomics Centre at SGUL.

### 2.2 Animals

Fourteen UK captive-bred female cynomolgus macaques (*Macaca fascicularis*), aged between 4 and 5 years at the beginning of the experiment, were housed in accordance with the Home Office (UK) Code of Practice (1989). Animals were sedated with ketamine hydrochloride prior to procedures. Menstrual cycles were determined by the onset of bleeding.

### 2.3 Immunisation

Animals were assigned to experimental groups (Table 1). Immunisation timings varied dependent upon individual menstrual cycles. Gp140 was formulated at 100 μg ml^−1^ in Carbopol gel as described previously [21]. 1 ml of the mixture was administered via a syringe inserted approximately 2 cm into the vagina. For any one cycle of intravaginal inoculation, animals received formulated product on 9 occasions at 2–3 daily intervals during the inter-menses phase of the menstrual cycle. For systemic immunisation, 100 μg gp140 was mixed with an equal volume of AS01 adjuvant and 0.2 ml injected into each deltoid muscle.

### 2.4 Genital tract secretions

Secretions were sampled using pre-weighed Weck-cel surgical spears (Medtronic Ophthalmics, Jacksonville, FL) placed either on the cervical os or the vaginal wall for 1 min. After reweighing, secretions were eluted from the sponges as described previously [21]. 8 μl of heat inactivated foetal calf serum was added to pooled extracts before freezing aliquots at −80 °C.

### 2.5 Total immunoglobulin and anti-gp140 binding antibody assays

Total immunoglobulin concentrations in mucosal eluates were measured by sandwich ELISA. 96-well plates (medium binding, Greiner Bio-One, Stonehouse, UK) were coated with either goat anti-monkey IgG (γ-chain-specific) (KPL, Gaithersburg, USA) or goat anti-monkey IgA (α-chain-specific) (KPL) at 2 μg ml^−1^. After washing and blocking, as detailed below for antibody ELISA, mucosal eluates were added at dilutions of 1/100 and 1/1000. Bound immunoglobulin was detected by addition of goat anti-monkey IgG (Fc-specific) HRP conjugate (AbD Serotec, Kidlington, UK) or goat anti-monkey IgA (Fc-specific) HRP conjugate. Standard curves were derived using purified rhesus monkey IgG (SouthernBiotech, Birmingham, USA) or purified human IgA (Sigma, UK) and concentrations in mucosal secretions calculated taking into account the dilution factor derived from the weight of sample. Due to the unavailability of purified monkey IgA, results for this isotype were expressed as units (U) ml^−1^.

Anti-gp140 binding antibodies were measured using a standardised ELISA. 96-well plates were coated with 50 μl per well of recombinant CN54gp140 at 5 μg ml^−1^ in PBS for 1 h at 37 °C. After washing four times in PBS containing 0.05% Tween 20 (PBS-T) reactive sites were blocked by incubation with PBS-T containing 10% foetal calf serum for 1 h at 37 °C. After further washing, serial dilutions of serum or mucosal eluates in PBS-T were added and incubated for 1 h at 37 °C. Bound IgG was detected with goat anti-monkey IgG (Fc-specific) HRP conjugate (Serotec) and bound IgA was detected with goat anti-monkey IgA (α-chain-specific) biotin conjugate (Acris, Herford, Germany) followed by avidin–HRP conjugate (Sigma). Normal control monkey serum was used as a negative control. Standard curves were derived using serum from a macaque immunised with HIV-1_W61D_ gp120 [28]. Antibody titres and concentrations of immunoglobulin were corrected for dilution factor derived from weight of sample/weight of sample + 600 assuming a density of 1 mg μl^−1^ [19].

### 2.6 Virus infectivity neutralisation assay

Neutralising antibody responses were measured against tier 1 and tier 2 HIV-1 envelope-pseudotyped viruses, prepared by transfection of 293T/17 cells, using a standardised luciferase-based assay in TZM.bl cells [29,30]. The 50% inhibitory concentration (IC_50_) titre was calculated as the dilution of serum that gave a 50% reduction in relative luminescence units (RLU) compared to the virus control wells after subtraction of cell control RLUs. Murine leukaemia virus (MuLV) negative controls were included in all assays.

### 2.7 Extraction of tissue mononuclear cells and ELISpot assay

Dissected spleen tissue and lymph nodes or marrow washed from the bone were dissociated in RPMI by sieving through a 100 μm mesh and then centrifuged at 4 °C for 10 min at 400 × *g*. Supernatant was removed and the pellet resuspended in residual media and washed once more with 10 ml RPMI. Cells were resuspended in 25 ml RPMI and were then filtered through a 50 μm filcon (BD Biosciences, Oxford, UK) before being layered onto Histopaque-1077 (Sigma, UK) and centrifuged at room temperature for 30 min at 1500 × *g*. Interface cells were collected and viable mononuclear cells counted.

*Ex vivo* amplified ELISpot assays were based on the method described by Bergmeier et al. [31]. PVDF membrane plates (Muliscreen HTSIP, Millipore) were treated with 35% ethanol for 1 min, washed three times with sterile PBS and coated with either recombinant CN54 gp140 or KLH (Calbiochem) at 10 μg ml^−1^ overnight at 4 °C. Following a further 6 washes with PBS-T, reactive sites were blocked by incubation with RPMI 1640 medium containing 10% FCS and pen/strep for 1 h at room temperature. Freshly recovered tissue MNCs were added to triplicate wells at 1 × 10^5^ and 5 × 10^5^ cells/well and incubated for 24 h at 37 °C in an atmosphere of 5% CO_2_. After further washing in PBS-T, bound secreted antibody was detected with either goat anti-monkey IgG-HRP (Serotec) diluted 1/2000 or with goat anti-monkey IgA-biotin (Acris) at 1/1000 followed by avidin–HRP (Sigma) diluted 1/2000. Spots were detected by addition of TMB substrate (Sureblue TMB 1-component peroxidise substrate, KPL) and enumerated with a reader. Total IgG and IgA ASC were assayed by the same method using plates coated with goat anti-monkey IgG (γ-chain-specific) (KPL) or goat anti-monkey IgA (α-chain-specific) (KPL) as capture antibodies.

### 2.8 Statistical analysis

Specified analyses were performed using SigmaPlot version 11 software.

## 3 Results

### 3.1 Repeated cycles of intravaginal immunisation primed a serum antibody response

Four cynomolgus macaques were inoculated intravaginally, each with 1 ml Carbopol gel containing 100 μg of CN54 gp140 on each of 9 occasions every 2 or 3 days during the inter-menses interval, followed by a further two cycles of intravaginal dosing and a final intramuscular immunisation with 100 μg of CN54 gp140 given in AS01 adjuvant (Group A: Table 1).

**Fig. 1:**
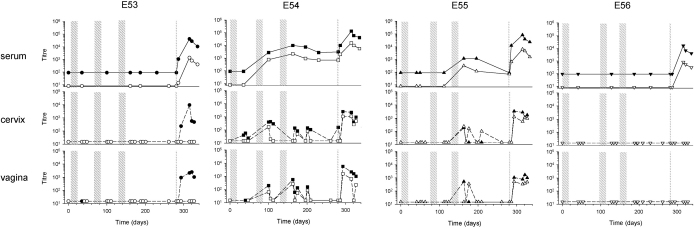
Serum and mucosally-detected anti-gp140 antibody titres after intravaginal immunisation followed by intramuscular immunisation (Group A). The intravaginal immunisation cycles, each consisting of 9× 100 μg gp140, are shown as broad stippled areas and intramuscular immunisation is indicated by a thin stippled line. For each animal, E53, E54, E55 and E56 results are shown for serum and cervical and vaginal fluids, with IgG indicated by filled symbols and IgA indicated by open symbols. Limits of detection for serum antibodies were titres of 100 and 10 for IgG and IgA respectively.

**Fig. 2:**
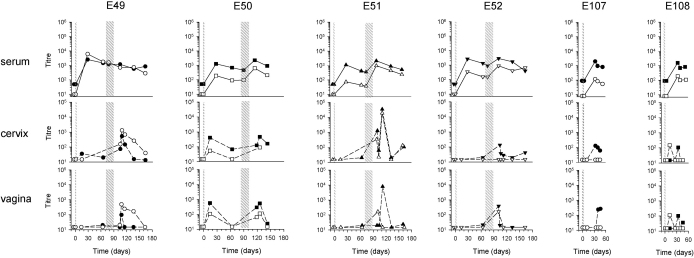
Serum and mucosally-detected anti-gp140 antibody titres after a single intramuscular immunisation followed by intravaginal immunisation (Group B) or a single intramuscular immunisation alone (Group D). The intramuscular immunisation is indicated by a thin stippled line and the intravaginal immunisation cycle, consisting of 9× 100 μg gp140, is shown as a broad stippled area. For each animal, E49, E50, E51, E52 (Group B) and E107 and E108 (Group D) results are shown for serum and cervical and vaginal fluids, with IgG indicated by filled symbols and IgA indicated by open symbols. Limits of detection for serum antibodies were titres of 100 and 10 for IgG and IgA respectively.

**Fig. 3:**
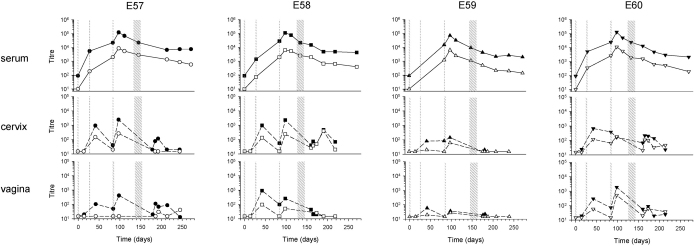
Serum and mucosally-detected anti-gp140 antibody titres after three intramuscular immunisations followed by intravaginal immunisation (Group C). The intramuscular immunisations are indicated by thin stippled lines and the intravaginal immunisation cycle, consisting of 9× 100 μg gp140, is shown as a broad stippled area. For each animal, E57, E58, E59 and E60 results are shown for serum and cervical and vaginal fluids, with IgG indicated by filled symbols and IgA indicated by open symbols. Limits of detection for serum antibodies were titres of 100 and 10 for IgG and IgA respectively.

All pre-treatment samples tested negative for gp140-specific IgG and IgA antibodies. Two animals of Group A mounted serum IgG and IgA anti-gp140 responses after multiple cycles of intravaginal immunisation: E54 after two cycles and E55 after 3 cycles (Fig. 1). IgG and IgA titres measured at the time of seroconversion (2800, 1200; IgG and 770, 320; IgA) fell within the range seen in sera from animals of Groups B, C and D following a single adjuvanted intramuscular immunisation (1110–5500; IgG and 75–6200; IgA) (Figs. 2 and 3). Titres were boosted in E54 after the third cycle of intravaginal immunisation and were similar to those measured in Group C after two adjuvanted intramuscular immunisations. In contrast, animals E53 and E56 did not seroconvert until given a final intramuscular immunisation. Of note however, peak titres of IgG measured in sera from all the Group A animals 34 days after intramuscular immunisation, regardless of prior seroconversion status, were consistently higher than those measured in Groups B, C and D after a single intramuscular immunisation [geometric mean titre (gmt) 51,880 versus 2198, *P* < 0.001; t-test]. Although the gmt serum IgA response was also higher (1778 versus 245) this difference did not reach statistical significance (*P* = 0.065; t-test). Interestingly, animal E53, despite lack of seroconversion following intravaginal immunisation, demonstrated anamnestic IgG and IgA antibody responses after intramuscular immunisation, with responses detected by 5 days.

Taken together these data indicate that non-adjuvanted, intravaginal immunisation can result in seroconversion and, when this does occur, IgG and IgA antibody titres are similar to those measured after a single adjuvanted intramuscular immunisation. Moreover, intravaginal immunisation in the absence of seroconversion can prime for a systemic memory response.

### 3.2 Anti-gp140 antibody was detected in female genital secretions following intravaginal vaccination only in animals that seroconverted

IgG and IgA antibodies were detected in cervical and vaginal samples intermittently from animals E54 and E55 following intravaginal immunisation. In general, antibodies were detected locally only upon seroconversion; however, in E54 IgG antibody was detected at low titres (24–58) in cervical samples after a single cycle of intravaginal immunisation and prior to seroconversion. In E55, IgG antibody was detected in both cervical and vaginal samples immediately upon seroconversion but not on 7 other occasions tested after seroconversion until intramuscular immunisation. IgA antibody was detected in cervical samples with titres ranging from 103 to 242 on 3 of 8 occasions tested but only on one occasion from vaginal samples. IgG and IgA antibodies were detected at a higher frequency in cervical and vaginal samples from E54 following intravaginal immunisation; however, titres fluctuated between positive peak values of IgG in the range 24–295 in cervical samples and 59–563 in vaginal samples to undetectable and similarly for IgA in the range 50–169 in cervical and 53–264 in vaginal samples to undetectable. There was no consistent pattern associating samples in which antibody was below the limit of detection with either the weight of the sample recovered or the total IgG or IgA content.

### 3.3 Intramuscular immunisation augmented mucosally-detected antibody responses in macaques primed intravaginally

Intramuscular immunisation of animals in Group A resulted in the appearance or the boosting of mucosally-detected antibodies in 3 of the 4 macaques. Furthermore, antibody titres were more stable than those seen after intravaginal immunisation alone over the study period (Fig. 1). Interestingly, in E53, where serum antibodies were undetectable before intramuscular boosting but showed an anamnestic response upon boosting, only IgG antibody was detectable locally despite total IgA concentrations of 2118–70,528 U ml^−1^ and 1338–28,838 U ml^−1^ in cervical and vaginal samples respectively (Table 2). The IgG antibody was unlikely due to blood contamination as in only one cervical sample was haemoglobin detected. In the two animals in which antibody had previously been detected mucosally both IgG and IgA antibody titres were boosted. In E54, peak titres for IgG antibody of 2500 and 5582 were detected in cervical and vaginal samples respectively compared to peak titres of 295 and 563 respectively prior to intramuscular boosting. Likewise IgA antibody peak titres of 1086 and 1522 were detected in cervical and vaginal samples respectively compared to peak titres of 169 and 264 respectively prior to intramuscular immunisation. Similarly in E55 peak titres for IgG antibody increased from 186 to 3360 and from 528 to 1719 in cervical and vaginal samples respectively and for peak titres of IgA from 242 to 1243 and from 355 to 515 respectively. Despite accelerated (anamnestic) serum responses following intramuscular boosting, in no case was a local anamnestic response detected. Animal E56 had no mucosally-detected antibody despite seroconversion; however, total IgG and IgA concentrations were consistently low in mucosal samples from this animal (Table 2). In contrast, IgG was usually detected in both cervical and vaginal samples from Group B animals following a single intramuscular immunisation when observed over a similar period of time (Fig. 2), but in any one animal this was irregular and overall at much lower titres than detected in animals E53, E54 and E55 that had received intravaginal priming (cervical gmt 63 versus 1298, and vaginal gmt 65 versus 1511; *P* < 0.001; Mann–Whitney rank sum test). Similarly, where detected, cervical and vaginal IgA titres were higher when intramuscular immunisation was preceded by intravaginal priming; however the small sample size precluded statistical analysis.

### 3.4 Serum and mucosally-detected anti-gp140 antibody responses were augmented by intravaginal immunisation of macaques primed with a single intramuscular immunisation

To determine the effect of priming/pre-existing antibody on intravaginal immunisation, 4 macaques received a single intramuscular immunisation 70–82 days prior to one cycle of intravaginal immunisation (Group B: Table 1) and a further 4 animals received 3 intramuscular immunisations followed by a single cycle of intravaginal immunisation 128–143 days after the last intramuscular immunisation (Group C: Table 1). Three of four animals of Group B had significantly higher serum IgG and IgA titres following intravaginal administration of gp140 (IgG *P* = 0.05, IgA *P* = 0.039; paired t test) (Fig. 2). In contrast, none of the animals of Group C had increased serum antibody following intravaginal administration (Fig. 3). As would be expected, titres of serum IgG and IgA were significantly higher at the time of intravaginal immunisation in animals of Group C that had received 3 intramuscular immunisations compared to those of Group B (IgG gmt: 18,197 versus 649, *P* < 0.001; IgA gmt: 1972 versus 173, *P* = 0.027; t-test).

Results for mucosally detectable antibody were more difficult to interpret given the variability seen at different sampling times and on some occasions between cervical and vaginal samples taken at the same time. All animals of Group B appeared to respond following intravaginal immunisation, including E49 that did not show a boost in serum antibody. This animal was unusual in that serum IgA titres were similar to IgG titres and IgA titres were higher than IgG titres in cervical and vaginal samples. Interestingly, total IgA concentrations were not elevated in cervical or vaginal secretions from this animal (Table 2) and significant haemoglobin contamination was only seen at Day 126, when titres of anti-gp140 IgA in the cervical sample had declined and were below the limit of detection in the vaginal sample.

Mucosally-detected antibody responses were seen in all animals of Group C following intramuscular immunisation. In most instances antibodies appeared following the second immunisation, subsequently waned and recovered following a further intramuscular exposure. For logistical reasons it was not possible to obtain mucosal samples immediately before intravaginal immunisation; however, antibodies were detected locally in all animals after the cycle of intravaginal immunisation but peak titres were not elevated. Overall, 3 intramuscular immunisations before intravaginal boosting conferred no advantage over a single intramuscular immunisation in terms of either the frequency or titre of antibody response detected in cervical and vaginal samples.

**Fig. 4:**
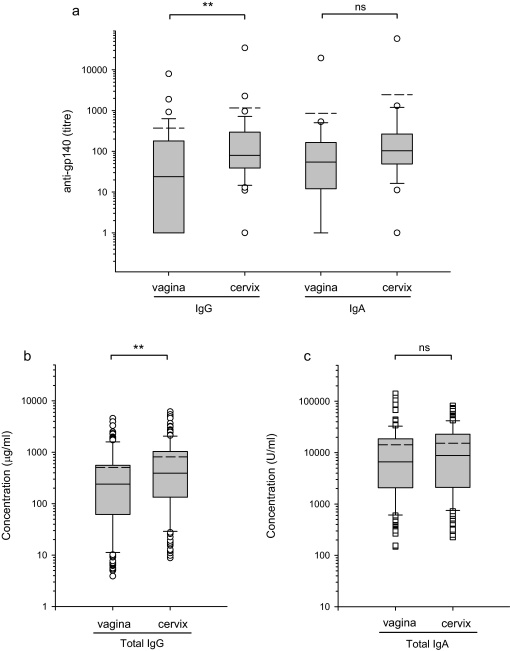
Comparison of anti-gp140 specific antibodies and total immunoglobulins in vaginal and cervical secretions. Animals were immunised either once or three times by intramuscular immunisation with gp-140 in AS01 adjuvant followed by a cycle of 9 intravaginal immunisations with gp140 in Carbopol gel (Groups B and C) (a) The median titres of IgG and IgA antibodies were significantly different at the *P* = 0.006 (**) level for IgG but were not significantly different (ns) for IgA *P* = 0.097 (Mann–Whitney ranked sum test). (b) Total IgG concentrations were significantly higher in cervical secretions, *P* = 0.001 (**) (Mann–Whitney ranked sum test). (c) Total IgA concentrations in fluids from the two sites were not significantly different, *P* = 0.22. Group mean (- - -) and median (—) titres are shown with box plots.

Overall in Groups C and D both IgG and IgA anti-gp140 antibody titres were higher in cervical fluids than vaginal fluids, with median titres of IgG of 80 and 24 and of IgA of 103 and 54 in vaginal and cervical samples respectively (Fig. 4). This difference however only reached statistical significance for IgG. Comparison for individual animals showed cervical samples to contain higher titre antibody than vaginal samples on 76% and 85% of occasions tested for IgG and IgA respectively. Likewise, total immunoglobulin concentrations were higher in cervical compared to vaginal samples (median IgG 392 μg ml^−1^ and 239 μg ml^−1^; median IgA 8752 U ml^−1^ and 6657 U ml^−1^ for cervix and vagina respectively) but only reached statistical significance for IgG (Fig. 4). Direct comparison of IgG titres with IgA titres in either site was not possible, as the IgA antibody assay used an additional amplification step that had previously been shown to give better discrimination between low positive results and background, non-specific binding. Comparison of total IgG and IgA concentrations was also precluded as a purified cynomolgus macaque IgA was unavailable for calibration of the IgA assay and therefore purified human IgA was used.

### 3.5 Serum neutralising activity against tier 1 envelope pseudotype correlated with binding antibody titre in animals primed intramuscularly and demonstrated restricted specificity

**Fig. 5:**
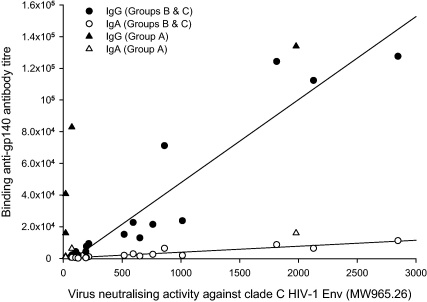
Correlation between virus neutralising activity and serum binding antibody titres. Neutralising activity was measured in sequential serum samples against tier 1 clade C HIV-1 MW963.26 pseudovirus and compared to serum IgG and IgA gp140 binding antibody titres from macaques immunised with gp140 by intravaginal-systemic (Group A) and systemic-intravaginal (Groups B and C) protocols.

Serum virus neutralising activity against clade C tier 1 MW965.26 pseudovirus was induced in 2 of 4 animals of Group A, albeit only at very low titre in one animal, following adjuvanted intramuscular immunisation; in 3 of 4 animals of Group B at low titre following intravaginal immunisation and in 4 of 4 animals of Group C following 3 intramuscular immunisations – this activity was not boosted by subsequent intravaginal immunisation. No activity was seen in animals of Group D 34 days after intramuscular immunisation (Table 3). In sera where neutralising activity was detected above the cut-off titre of 60, strong correlations were found between this activity and both IgG (*r* = 0.87, *P* < 0.001) and IgA (*r* = 0.82, *P* < 0.001; Pearson product moment correlation) anti-gp140 binding titres (Fig. 5). In sera from animals of Groups B and C, anti-gp140 IgG titres greater than 3000 were invariably predictive of neutralising activity. Notably, this was not the case for Group A, where despite the induction of high titres of anti-gp140 IgG (16,000–134,000) following intravaginal immunisation, appreciable neutralising activity was detected only in animal E54 which had the highest binding antibody titre.

To determine the breadth of neutralising activity, sera were tested against a range of pseudotypes including 4 other tier 1 envelopes. Although no activity was seen against TV1.21, another clade C envelope, some activity was detected against the clade B SF162.LS (Table 3), but not against clade B, BaL.26 or clade A, DJ263.8. Neither was any neutralising activity seen against any of 13 tier 2, clade C envelopes (96ZM651.02, Du156.12, Du172.17, Du422.1, CAP45.2.00.G3, CAP210.2.00.E8, ZM197M.PB7, ZM214M.PL15, ZM233M.PB6, ZM249M.PL1, ZM53M.PB12, ZM109F.PB4, ZM135M.PL10a). Cross-reactivity between clade C and clade B was restricted to sera with high-titre neutralisation against MW965.26 (titres of 594–2846); however sera from animal E58, with titres within this range failed to cross-react.

### 3.6 High frequencies of *ex vivo* IgG antibody secreting cells were detected in iliac lymph nodes of intravaginally-primed macaques that seroconverted only after intramuscular immunisation

To determine the distribution of *ex vivo* anti-gp140 specific antibody secreting cells (ASC), mononuclear cells (MNC) were obtained from tissues of Groups A and D animals at necropsy. Insufficient cells were recovered from vagina and cervix; but MNC were recovered from spleen, bone marrow, interior iliac lymph nodes, mesenteric lymph nodes and axillary lymph nodes. Frequencies of ASC ranged from 52 to 1065 sfu/10^6^ MNC for total IgG and from 115 to 906 sfu/10^6^ MNC for total IgA in all tissues other than bone marrow, where frequencies for both isotypes exceeded 2500/10^6^ MNC (Table 4). In most instances, only low frequencies of anti-gp140 ASC were detected; notably however, IgG anti-gp140-specific ASC represented 6% and 16% of total IgG secreting cells recovered from the interior iliac lymph nodes of animals E53 and E56 respectively; the animals that failed to seroconvert after intravaginal immunisation but responded following intramuscular immunisation (Table 4).

## 4 Discussion

This is the first demonstration that intravaginally delivered soluble recombinant HIV-1 gp140 is immunogenic in primates in the absence of a conventional mucosal adjuvant. Although intravaginal immunisation alone was less efficient in macaques compared to in rabbits at inducing serum and mucosally-detected antibodies, where a single cycle of immunisation was sufficient to induce responsiveness [21], women in a parallel clinical trial, were also essentially unresponsive following a single cycle of immunisation (Lewis et al., personal communication). This may reflect inefficiency in accessing sites of inductive immunity, despite the strategy of repeated inoculation designed to increase the likelihood of exposure to immunogen during the menstrual cycle. However, there may only be a narrow window for induction of immune function during any one cycle as shown by studies in women [19]. It was therefore encouraging that two macaques responded with serum and mucosally-detected responses after multiple cycles of intravaginal administration and that a third macaque was primed for a response as revealed by subsequent intramuscular immunisation. This “silent priming” of a memory B-cell response may be similar to that observed in macaques following mucosal exposure to “subinfectious” doses of SIV where antigen-primed B cells were detected *in vitro* by amplified ELISpot in the absence of seroconversion [32]. In the present study, for logistical reasons it was not possible to sample lymphoid tissues over time; intriguingly however, high frequencies of gp140-specific IgG ASC were detected *post mortem* in the interior iliac lymph nodes from the two intravaginally immunised macaques that seroconverted only after intramuscular immunisation. These lymph nodes, which drain both the female genital and the rectal tracts, have been associated with protective immunity in SIV challenge studies [33]. In the present study we were unable to recover sufficient cells directly from cervico-vaginal tissues for ELISpot analysis and therefore assessment of the frequency of mucosal ASC was not possible. However, the appearance and apparent sustainability of mucosally-detected antibodies after intramuscular boosting was encouraging and suggested that immune cells may be homing to local tissue. Further *in vivo* experiments are needed to establish the longevity and functional activity of the mucosally-detected antibody. How mucosal immunisation primes for a systemic boost is unknown and suggests that mucosally-primed B cells may cross-over between compartments and/or that vaginally-administered antigen reaches both systemic and mucosal sites of inductive immunity.

Conversely, the mechanism by which intramuscular immunisation primes antigen recognition following intravaginal exposure is not established; however, the dose and secondary signalling requirements for memory B-cell activation are less stringent than for B cell priming. Presumably, despite the systemic route of priming, at least some memory cells migrate to the female genital tract. It is also conceivable that antibody induced by intramuscular priming complexes to vaginally applied antigen and facilitates uptake and presentation by Fc receptor-bearing APC. The enhanced immunogenicity of immune complexes in general when administered systemically is well documented and has recently been reported for HIV-1 gp120 [34]; however, there is a paucity of data regarding mucosal routes [35]. The lack of vaginal boosting of serum responses in macaques that had received 3 intramuscular immunisations may simply be a saturation effect; however, the lack of local antibody boosting was disappointing and suggests that there may be downmodulation of local memory in the presence of high levels of systemic immunity.

Taken together, the results suggest that the concentration of gp140 used for intravaginal immunisation may have been below the threshold required for efficient stimulation of an antibody response *de novo* from the precursor B-cell pool and at the threshold for boosting a memory response. It would now be interesting to determine if higher doses of non-adjuvanted gp140 would be more effective. Furthermore, although formulating gp140 in rheologically structured vehicles designed to enhance antigen retention in the vaginal vault appeared to offer little advantage over Carbopol formulation in rabbits [36] such vehicles may be more beneficial in non-human primates and humans, where access to the immune system via this route may be more restricted.

The mechanisms responsible for antibody appearance in cervical and vaginal fluids are yet to be fully defined. Some antibody is derived from plasma by transudation and some may be produced locally. Indeed, testing of secretions from 6 macaques, where volume allowed, revealed IgA anti-gp140 containing secretory component (data not shown). For technical reasons it was not possible to directly compare total and specific IgG and IgA levels however others have reported that, as in women [37], IgG is the predominant immunoglobulin in the lower female genital tract of macaques [38] and IgG as well as IgA ASC are present in macaque vaginal tissues [39]. Also, as in women [40], both total and specific IgG and IgA were found to be higher in cervical samples than in vaginal. The wide variation in local immunoglobulin and antibody levels for any individual animal may have been due to the effects of the menstrual cycle as reported in macaques and women [41,42,38]; however, the present study was not powered to analyse this variable.

An effective vaccine will require not only sustained antibody production into mucosal fluids but the antibodies will need to have potent and broad virus neutralising activity. It is known that monomeric gp120 generally fails to elicit such activity [43–46] and for this reason we used a trimeric envelope immunogen, gp140, that has demonstrated remarkable stability *in vitro* (D. Katinger, personal communication) and is therefore more likely to mimic the native virion envelope spike [2]. Although cross-clade neutralising activity was restricted to MW965.26 and clade B SF162.LS envelope-bearing pseudoviruses and disappointingly no activity was seen against any of a broad range of clade C envelopes, this study has shown that this narrow specificity is not exclusively due to formulation of the immunogen in Carbopol and/or the vaginal route of administration, as similar results were obtained after intramuscular immunisation in the presence of AS01 adjuvant. Moreover, as in rabbits [21], serum antibodies did not recognise the highly immunogenic gp41-ED residues 598–597 [47] (data not shown), suggesting that the gp41 region of the molecule may be occluded possibly because of the lack of membrane anchoring. Interestingly macaques have been protected against vaginal challenge with SHIV_SF162_ following systemic or nasal/systemic immunisation with HIV-1_SF162_ ΔV2 gp140 and protection was associated with serum neutralising antibody [48].

Although the restricted serum neutralising activity obtained is of questionable relevance for a protective HIV-1 vaccine it is interesting that the correlation between anti-gp140 IgG binding antibody titre and neutralising activity seen in animals that were primed intramuscularly did not hold true for animals primed intravaginally. This observation suggests factors other than antibody titre alone may be important, including antibody subclass, avidity and fine specificity. Furthermore, we were unable to measure neutralising activity in mucosal fluids and there is a clear need for the development of micro-neutralisation assays that can be used with small volumes of biological fluid.

The results obtained here inform the design of our next clinical trial that will run in parallel with a “paraclinical” macaque study that will include envelope-SHIV challenge. Through this iterative process it will be possible to cross-validate the macaque model – essential for the identification of correlates of protective immunity. Moreover, although we cannot discount the possibility that repeated sedation of the macaques may have influenced immune responsiveness, our results support the idea that mucosal exposure to HIV, either at a subinfectious dose or in a “protected” form, for example in the presence of a microbicide, may modulate specific immunity and may augment vaccination.

## Figures and Tables

**Table 1:** Assignment of animals to experimental groups.

Group	Macaque numbers	Day of immunisation^a^	Route of immunisation
A	E53, E54, E55, E56	0–21	ivag^b^ 9×
		63–117	ivag 9×
		131–170	ivag 9×
		281	im^c^
B	E49, E50, E51, E52	0	im
		70–101	ivag 9×
C	E57, E58, E59, E60	0	im
		28	im
		84	im
		128–161	ivag 9×
D	E107, E108	0	im

a For any one animal intravaginal (ivag) immunisation was done 9 times over a 19-day interval; the exact start times varied depending upon the menstrual cycle.

b Each ivag dose consisted of 100 μg gp140 formulated in Carbopol gel.

c Intramuscular immunisation consisted of 100 μg gp140 formulated in AS01 adjuvant.

**Table 2:** Total IgG and IgA concentrations in cervical and vaginal samples of each macaque.

Group	Animal no.	Total immunoglobulin concentration, median (range)			
		IgG (μg/ml)		IgA (U/ml)	
		Cervical	Vaginal	Cervical	Vaginal
A	E53	683 (152–6156)	474 (99–2435)	11,654 (2118–70,528)	9152 (1338–28,838)
	E54	909 (218–2184)	493 (90–1788)	28,490 (6186–64,736)	19,920 (8525–116,965)
	E55	653 (223–3650)	315 (37–1925)	27,790 (8752–81,942)	28,143 (4211–141,878)
	E56	28 (9–112)	9 (5–114)	933 (400–3770)	619 (265–4018)
B	E49	100 (15–309)	51 (4–201)	938 (300–15,939)	1512 (148–13,300)
	E50	2754 (1140–5429)	1785 (432–4579)	4832 (1935–6704)	4587 (763–13,552)
	E51	191 (42–4949)	634 (39–2486)	4366 (799–49,625)	5328 (1607–44,455)
	E52	216 (66–1638)	272 (72–966)	3345 (724–17,451)	2821 (1584–14,805)
C	E57	148 (10–710)	69 (13–147)	1594 (225–7341)	1214 (286–1525)
	E58	392 (22–1750)	82.6 (6–434)	10,245 (1562–44,098)	4134 (487–22,940)
	E59	140 (38–529)	174 (26–1853)	6711 (52–16,459)	5459 (630–18,113)
	E60	262 (90–1708)	92 (47–444)	5597 (1265–33,572)	4388 (408–18,585)
D	E107	2295 (234–4592)	1167 (294–1991)	15,340 (8059–29,253)	17,225 (3919–26,241)
	E108	1378 (314–2478)	924 (341–2261)	7269 (4703–27,368)	9740 (4702–85,936)

**Table 3:** Serum virus neutralising activity.

Animal No.	Day 0 (pre-imm.)				Day 196 (26–47 days post 3× ivag)				Day 281 (111–132 days post 3× ivag)				Day 315 (34 days post 1× i.m.)			
	VNA		Binding Ab		VNA		Binding Ab		VNA		Binding Ab		VNA		Binding Ab	
	Clade C (MW965)	Clade B (SF162)	IgG	IgA	Clade C (MW965)	Clade B (SF162)	IgG	IgA	Clade C (MW965)	Clade B (SF162)	IgG	IgA	Clade C (MW965)	Clade B (SF162)	IgG	IgA
(a) Group A																
E53	<60	<60	<100	<10	NT	NT	<100	<10	<60	<60	<100	<10	<60	<60	40,800	1370
E54	<60	<60	<100	<10	<60	<60	7600	940	<60	<60	3100	710	**1980**	**151**	134,000	16,000
E55	<60	<60	<100	<10	<60	<60	1200	120	288^a^	74^a^	<100	70	**72**	<60	82,900	6300
E56	<60	<60	<100	<10	NT	NT	<100	<10	<60	<60	<100	<10	<60	<60	16,000	800

Animal No.	Day 0 (pre-imm.)				Day 64 (64 days post 1× i.m.)				Day 99–115 (9–14 days post 1× ivag)				Day 199 (98–110 days post 1× ivag)			
	VNA		Binding Ab		VNA		Binding Ab		VNA		Binding Ab		VNA		Binding Ab	
	Clade C (MW965)	Clade B (SF162)	IgG	IgA	Clade C (MW965)	Clade B (SF162)	IgG	IgA	Clade C (MW965)	Clade B (SF162)	IgG	IgA	Clade C (MW965)	Clade B (SF162)	IgG	IgA
(b) Group B																
E49	<60	<60	<100	<10	<60	<60	1470	1810	**65**	<60	1200	720	<60	<60	450	190
E50	<60	<60	<100	<10	<60	<60	690	91	**74**	<60	2260	635	<60	<60	700	140
E51	<60	<60	<100	<10	<60	<60	420	45	**115**	<60	2130	980	<60	<60	400	200
E52	<60	<60	<100	<10	<60	<60	1330	156	<60	<60	2840	900	<60	<60	540	190

Animal No.	Day 0 (pre-imm.)				Day 98 (14 days post 3× i.m.)				Day 134–148 (36–50 days post 3× i.m.)				Day 160–179 (13–22 days post 1× ivag)				Day 270–272 (111–127 days post 1× ivag)			
	VNA		Binding Ab		VNA		Binding Ab		VNA		Binding Ab		VNA		Binding Ab		VNA		Binding Ab	
	Clade C (MW965)	Clade B (SF162)	IgG	IgA	Clade C (MW965)	Clade B (SF162)	IgG	IgA	Clade C (MW965)	Clade B (SF162)	IgG	IgA	Clade C (MW965)	Clade B (Sf162)	IgG	IgA	Clade C (MW965)	Clade B (SF162)	IgG	IgA
(c) Group C																				
E57	<60	<60	<100	<10	**1814**	**453**	12,4400	8700	**594**	**121**	22,700	2900	**159**	<60	NT	NT	**196**	<60	7600	590
E58	<60	<60	<100	<10	**2128**	<60	112,400	6400	**761**	<60	21,500	2560	**517**	<60	15,200	1950	**192**	<60	4300	400
E59	<60	<60	<100	<10	**860**	**73**	71,200	6400	**216**	<60	9360	1100	**105**	<60	4300	510	<60	<60	2000	140
E60	<60	<60	<100	<10	**2846**	**96**	127,600	11,200	**1012**	**69**	23,800	1880	**651**	<60	13,000	1550	**125**	<60	2100	190

Animal No.	Day 0 (pre imm.)				Day 34 (34 days post i.m.)			
	VNA		Binding Ab		VNA		Binding Ab	
	Clade C (MW965)	Clade B (SF162)	IgG	IgA	Clade C (MW965)	Clade B (SF162)	IgG	IgA
(d) Group D								
E107	<60	<60	<100	<10	<60	<60	2000	120
E108	<60	<60	<100	<10	<60	<60	1600	190

Bold values indicate specific neutralisation with no background activity on MuLV pseudotype virus.

a Non-specific activity – VNA titre of 640 against MuLV pseudotype virus.

**Table 4:** Distribution of gp140-specific antibody secreting cells after intramuscular alone and intramuscular (1×) and intravaginal immunisation.

	Frequency of antibody secreting cells: gp140-specific (total): sfu/10^6^											
	E53		E54		E55		E56		E107		E108	
	IgG	IgA	IgG	IgA	IgG	IgA	IgG	IgA	IgG	IgA	IgG	IgA
Spleen	0 (995)	0 (101)	4 (120)	1 (496)	0 (1229)	0 (130)	12 (99)	0 (637)	4 (52)	0 (553)	0 (0)	0 (643)
Bone M	0 (≫)	2.5 (≫)	42 (≫)	22 (≫)	0 (≫)	12 (≫)	2 (≫)	1 (≫)	5 (≫)	12 (≫)	0 (≫)	1 (≫)
Iliac LN	41 (672)	0 (397)	7 (665)	5 (415)	0 (343)	0 (332)	75 (470)	0 (197)	0 (731)	0 (906)	2 (971)	0 (528)
Mes LN	0 (946)	0 (577)	0 (787)	0 (736)	0 (451)	2 (298)	0 (1065)	0 (563)	0 (795)	2 (821)	0 (875)	0 (835)
Ax LN	0 (706)	0 (357)	0 (0)	1 (307)	0 (306)	0 (115)	0 (356)	0 (141)	0 (325)	0 (275)	0 (523)	0 (409)
